# Noncommunicable diseases: stepping up the fight

**DOI:** 10.2471/BLT.15.030115

**Published:** 2015-01-01

## Abstract

The prevention and control of noncommunicable diseases is now a top priority in many countries. Sergey Boytsov tells Fiona Fleck how the Russian Federation is collaborating with other Commonwealth of Independent States’ countries.

Q: Health officials gathered last month to launch a project for the prevention and control of noncommunicable diseases in WHO’s European Region that will lead to the opening of a WHO [geographically-dispersed office] in Moscow. What will the project do? 

A: This project is very important for the European Region. The Russian Federation has provided a grant of US$ 22 million to the WHO Regional Office for Europe over the next five years to build capacity in the European region to address the epidemic of noncommunicable diseases – cardiovascular disease, cancers, diabetes and chronic respiratory disease – that are the main killers in these countries. That means, among other things, providing technical support and training so that countries are able to draw up their own national plans to prevent and control these diseases. 

Q: How are Russian centres collaborating with other Commonwealth of Independent States’ (CIS) countries, which were once part of the Soviet Union, on this challenge? 

A: The goal of our collaboration is to reduce smoking, to make peoples’ diets healthier, to stop alcohol abuse and make people more physically active in our countries by exchanging information and sharing experiences on NCDs, building international networks and through data collection and analysis of cardiovascular and other diseases. Our centre – the National Research Centre for Preventive Medicine – is organizing collaborative international epidemiological projects with Kazakhstan and Kyrgyzstan and will soon do so with Armenia and Georgia. We are running international seminars on oncological or cardiovascular screening with Belarus and Kazakhstan. In addition, we are providing technical support to other countries. For example, we are translating WHO documents into the Russian language, such as resolutions and conference declarations, as Russian is spoken not only here in the Russian Federation but in other CIS countries. This collaboration is guided by the Global Action Plan for the prevention and control of NCDs 2013–2020 which is a great document – not just a text book – as it provides practical advice along with a timetable, indicators and goals. In addition, Moscow State Medical University is providing training on the prevention and control of these diseases and the first course was held in February last year for 25 health officials from Central Asian and east European countries. The university also provides continuing medical education for physicians from CIS countries. On the international level, our country has proposed a second global ministerial conference on healthy lifestyles and NCDs, to be held in Moscow in 2016.

Q: When did noncommunicable diseases start to become a major health problem in the Russian Federation?

A: The increase in premature mortality from these diseases is associated with the second wave of urbanization in our country. The first was in the 1930s. The second started in the 1960s, when people migrated from rural areas to cities and started to eat foods with a higher fat and salt content. These changes, combined with a reduction in physical activity, resulted in a higher prevalence of hypertension (high blood pressure) and atherosclerosis (clogged arteries). So, in the 1960s, premature mortality associated with NCDs started to increase and these increases accelerated during the 1980s and 1990s. 

Q: When did NCDs become a priority for the Russian and other CIS governments?

A: The medical and scientific communities, as well as policy-makers, have long been familiar with research and WHO recommendations on these diseases and were fully aware of the levels of mortality from these diseases in our country. But although premature mortality from these diseases continued to grow in the 1990s, routine prevention and control of these diseases was not really possible because of social and economic upheaval. Today it’s hard to imagine why it was so difficult to take systematic measures to address these diseases at that time. At the end of the 1990s we started to pay more attention to the problem and ran a successful pilot project, as part of the WHO’s Countrywide Integrated Noncommunicable Diseases Intervention programme, which allowed us to demonstrate the effectiveness of a prevention programme in several regions. Also, a federal programme launched in 2002–3 to address hypertension has also been successful in reducing the prevalence of high blood pressure. Inspired by these successes, our policy-makers decided to take systematic measures in 2009 and started to establish 500 health centres for adults and 200 for children across the country.

“Today it’s hard to imagine why it was so difficult to take systematic measures to address NCDs at that time.”

Q: What other measures have Russian policy-makers taken to address the NCDs epidemic? 

A: In 2010, a health protection law was passed on the prevention and control of these diseases. In 2011, Moscow held the first global ministerial conference on healthy lifestyles and NCDs in which ministers from many countries participated. For our country, it became clear that the development of health policy to prevent and control these diseases could only be done on a multisectoral level and so the next step was the preparation and acceptance of a state programme for health development, which was adopted in 2012. The first part of this programme is prevention of NCDs and educating people about lifestyle risk factors, with clearly defined criteria and indicators for its implementation on the federal level. From this, regional programmes have been developed.

*Q: Can you tell us about the new system of health check-ups, called *dispanserizatsiya* (*диспансеризация*), introduced recently in the Russian Federation to combat NCDs?*

A: It is a two-stage process that applies to all adults aged over 21 years. The first stage is a check-up to identify risk factors for noncommunicable diseases. The second stage involves further investigations and tests to confirm any suspected diagnoses, for example, of a cancer or of diabetes. The physician is required to contact the patient if there is a problem, so patients are not lost to follow up. Physicians are refunded for the check-ups by the mandatory health insurance. 

Q: How is it going? 

A: In 2013, when we started the programme, 21 million adults across the whole country received check-ups under the new system. More than 1.8 million of them were diagnosed with cardiovascular disease and more than 27 000 were found to have some form of cancer. By the end of November 2014, a further 20 million adults had passed through the new system. 

Q: What do you say to critics who argue that too few cases are detected by such a huge programme, and that the costs outweigh the benefits? 

A: Above all, I would stress that considering the high level of premature mortality from NCDs in our country, these measures are essential. One of the most important results of the new system of health check-ups is that we’re not only detecting people who already have these diseases, but those with a high risk of developing cardiovascular disease, constituting about 23% of the adult population. By addressing these risk factors early enough through lifestyle changes, we can significantly reduce premature mortality from these diseases. Of course we do not expect the new system to produce cost–benefits after one year. The earliest point at which we may see cost–benefits would be in four years’ time, once we have completed the first cycle of three years and most of the adult population has passed through the system.

“Considering the high level of premature mortality from NCDs in our country, these measures are essential.”

Q: What challenges did you face while rolling out the new system?

A: Much effort, particularly on the part of nurses and medical assistants, has gone into educating people and explaining why regular check-ups are necessary. Initially, we were afraid that physicians would be overwhelmed. When you think that there can be as many as 1800 to 2700 people affected by the programme per physician in some regions – it’s quite a challenge. But as recommended by the Federal Ministry of Health, they are dividing the adult population in their districts into three age groups, each of which is invited to come for a free check-up every three years. So our experience is reassuring. The check-ups are not mandatory, but recommended. We are working closely with the media to explain why, while emphasizing that these check-ups are free of charge.

Q: The government enforced tobacco control laws recently, what more can be done in terms of new laws and regulations to improve people’s health in other areas?

A: Tobacco consumption has been falling in our country over the last four years, judging by falling sales of cigarettes, and epidemiological research shows that these measures have reduced smoking by 5–7%. The new law came into force in 2013, so it’s early to assess the effect, but it should soon reinforce the downward trend for tobacco consumption. More could be done in terms of regulations regarding reductions in fat, sugar and salt content of processed foodstuffs. These issues were once the domain of experts, but now deputies of the State Duma (lower house of parliament) and other decision-makers are discussing these issues too. 

Q: What is being done to reduce the harmful consumption of alcohol?

A: Certainly, alcohol abuse is a significant risk factor for premature deaths particularly among men under the age of 60. In addition to restrictions on the advertising of alcoholic beverages and raising excise taxes, there are important efforts to create new jobs as this problem is often associated with unemployment. Since we launched the new system of health check-ups, we have identified about 300 000 people who are at risk of alcohol abuse and who are undergoing in-depth preventive counselling.

Q: What are the next steps in the new NCDs project in your country? 

A: We need to develop communication strategies to motivate people to take better care of their health to give them longer lives. We need to work with many other sectors – agriculture, transport and education – to create an environment that promotes healthy lifestyles. We must implement the many tried and tested strategies for preventing these diseases and their risk factors, while monitoring the health of every citizen through the new system of check-ups. It’s encouraging that we have made progress in our country in terms of tackling these diseases, but it’s still early days. The hard work must continue and hopefully in coming years we’ll see further improvements. 

